# Improved neurodevelopmental prognostication in isolated corpus callosal agenesis: fetal magnetic resonance imaging‐based scoring system

**DOI:** 10.1002/uog.22102

**Published:** 2021-07-01

**Authors:** M. C. Diogo, S. Glatter, D. Prayer, G. M. Gruber, D. Bettelheim, M. Weber, G. Dovjak, R. Seidl, G. Kasprian

**Affiliations:** ^1^ Department of Radiology, Division of Neuro‐ and Musculoskeletal Radiology Medical University of Vienna Vienna Austria; ^2^ Neuroradiology Department Hospital Garcia de Orta Almada Portugal; ^3^ Department of Pediatrics and Adolescent Medicine, Division of Pediatric Neurology Medical University of Vienna Vienna Austria; ^4^ Department of Anatomy and Biomechanics Karl Landsteiner University of Health Sciences Krems Austria; ^5^ Department of Gynecology and Obstetrics Medical University of Vienna Vienna Austria

**Keywords:** corpus callosal agenesis, corpus callosum, fetal MRI, neurodevelopmental outcome, prenatal diagnosis

## Abstract

**Objectives:**

Corpus callosal agenesis (CCA) is one of the most common brain malformations and is generally associated with a good outcome when isolated. However, up to 25% of patients are at risk of neurodevelopmental delay, which currently available clinical and imaging parameters are inadequate to predict. The objectives of this study were to apply and validate a fetal magnetic resonance imaging (MRI) anatomical scoring system in a cohort of fetuses with isolated CCA and to evaluate the correlation with postnatal neurodevelopmental outcome.

**Methods:**

This was a retrospective cohort study of cases of prenatally diagnosed isolated CCA (as determined on ultrasound and MRI), with normal karyotype and with known postnatal neurodevelopmental outcome assessed by standardized testing. A fetal brain MRI anatomical scoring system based on seven categories (gyration, opercularization, temporal lobe symmetry, lamination, hippocampal position, basal ganglia and ventricular size) was developed and applied to the cohort; a total score of 0–11 points could be given, with a score of 0 representing normal anatomy. Images were scored independently by two neuroradiologists blinded to the outcome. For the purpose of assessing the correlation between fetal MRI score and neurodevelopmental outcome, neurodevelopmental test results were scored as follows: 0, ‘below average’ (poor outcome); 1, ‘average’; and 2, ‘above average’ (good outcome). Spearman's rank coefficient was used to assess correlation, and inter‐rater agreement in the assessment of fetal MRI score was calculated.

**Results:**

Twenty‐one children (nine females (42.9%)) fulfilled the inclusion criteria. Thirty‐seven fetal MRI examinations were evaluated. Mean gestational age was 28.3 ± 4.7 weeks (range, 20–38 weeks). All fetuses were delivered after 35 weeks' gestation with no perinatal complications. Fetal MRI scores ranged from 0 to 6 points, with a median of 3 points. Inter‐rater agreement in fetal MRI score assessment was excellent (intraclass correlation coefficient, 0.959 (95% CI, 0.921–0.979)). Neurodevelopmental evaluation was performed on average at 2.6 ± 1.46 years (range, 0.5–5.8 years). There was a significant negative correlation between fetal MRI score and neurodevelopmental outcome score in the three areas tested: cognitive (ρ = –0.559, *P* < 0.0001); motor (ρ = –0.414, *P* = 0.012) and language (ρ = –0.565, *P* < 0.0001) skills. Using fetal MRI score cut‐offs of ≤ 3 (good outcome) and ≥ 4 points (high risk for poor outcome), the correct prognosis could be determined in 20/21 (95.2% (95% CI, 77.3–99.2%)) cases.

**Conclusion:**

By assessing structural features of the fetal brain on MRI, it may be possible to better stratify prenatally the risk of poor neurodevelopmental outcome in CCA patients. © 2020 Authors. *Ultrasound in Obstetrics & Gynecology* published by John Wiley & Sons Ltd on behalf of International Society of Ultrasound in Obstetrics and Gynecology.

**CONTRIBUTION**

**What are the novel findings of this work?**

The proposed fetal magnetic resonance imaging (MRI) scoring system for risk stratification in cases of corpus callosal agenesis is reliable and easy to apply. Combining different morphological MRI features of the fetal brain facilitates prenatal risk stratification for poor neurodevelopmental outcome in corpus callosal agenesis.

**What are the clinical implications of this work?**

Application of the proposed fetal MRI scoring system could lead to improved prognostication and risk stratification and more precise counseling of patients with a prenatal diagnosis of corpus callosal agenesis.

## INTRODUCTION

Corpus callosal agenesis (CCA) involves the congenital absence of all or part of the CC and is one of the most common central nervous system (CNS) malformations^1^, ^2^, ^3^, ^4^. The CC is the main commissural pathway in the human forebrain. All structural parts of the human CC are formed by 18 weeks' gestation^5^; however, it reaches full maturity only in late adolescence^5^. The complex process of CC formation may be disturbed by a variety of genetic, toxic, metabolic and disruptive processes^1^. Despite state‐of‐the‐art work‐up, the background of CCA remains unclear in up to 70% of cases^1^, ^6^, ^7^.

The CC is paramount in the transmission and integration of sensory, motor and cognitive information^8^, ^9^, ^10^. Even in the absence of associated conditions, CCA can manifest with a wide spectrum of cognitive, behavioral and neurological consequences^11^, ^12^. Although development is within the normal range in 70–88% of so‐called isolated CCA cases, the risk of severe neurodevelopmental delay exists^9^, ^13^, ^14^, ^15^, ^16^, ^17^. This risk seems to be independent of neuroanatomical profile and whether the agenesis is complete or partial^16^, ^18^, rendering current imaging techniques inadequate at correctly identifying high‐risk cases and making prenatal counseling on an individual basis challenging. This consequently leads to uncertainty and anxiety amongst parents‐to‐be^19^.

Although CCA can be detected on first‐trimester ultrasound (US)^20^, fetal magnetic resonance imaging (MRI) provides improved diagnosis and identification of

associated anomalies^15^, ^21^ and may help characterize fiber tracts and their altered connectivity using diffusion‐tensor imaging (DTI)^22^.

Determining if CCA is isolated is not straightforward, as there is no standard definition. In patients with CCA, several related abnormal anatomical and structural features may be found prenatally, such as colpocephaly, enlarged atrial size or changes in hippocampal volume^4^, ^9^, ^23^. While *per se* these do not constitute malformations, their presence, particularly if found in association, could be a manifestation of globally altered brain development with poorer function and a higher chance of an underlying genetic background. Quantifying these changes may help optimize prenatal assessment.

The aims of this study were to develop a fetal MRI‐based scoring system for isolated CCA, including the aforementioned anatomical features, in an attempt to further differentiate neurodevelopmental outcome groups, and to evaluate retrospectively its correlation with neurodevelopmental outcome.

## SUBJECTS AND METHODS

### Fetal MRI scoring system

A neuroimaging expert (G.K.) designed a fetal MRI scoring system for prenatal imaging assessment of isolated CCA cases, based on anatomical fetal brain features and previous cumulative insights into the prenatal phenotype of CCA^21^, ^22^, ^23^, ^24^. It consists of seven categories (gyration^25^, ^26^, ^27^, ^28^, ^29^, opercularization^30^, temporal lobe (a)symmetry^24^, lamination^31^, ^32^, ^33^, hippocampal abnormalities^23^, ^28^, basal ganglia and ventricular enlargement) scored 0–2 points (Table 1), with a maximum attainable score of 11 points. A detailed description of each parameter and the reasons for its inclusion in the scoring system can be found in Appendix S1, and examples can be seen in Figures S1–S9.

**Table 1:** Magnetic resonance imaging scoring system for fetuses with corpus callosal agenesis

Parameter	Score (points)		
	0	1	2
Gyration	Normal	Mildly delayed (≤ 2 weeks)	Delayed (> 2 weeks)
Opercularization	Normal	Delayed	—
Temporal lobe asymmetry*	Asymmetrical (R > L)	Symmetrical (R = L) or inverted (L > R)	—
Hippocampi	Normal	Malrotation (mild to moderate and/or unilateral)	Verticalization (bilateral), reduced volume
Lamination	Normal	—	Abnormal
Basal ganglia	Normal	Abnormal	—
Ventricular size†	Normal (< 10 mm)	10–14.9 mm	≥ 15 mm

* Temporal lobe asymmetry described according to Kasprian *et al*.^24^; if not assessable (i.e. at later gestational ages (> 32 weeks)), a score of 0 should be given.

† Measured at level of atrium; if ventricular size is asymmetrical, larger ventricle should be assessed. L, left; R, right.

### Patients and setting

In order to validate the fetal MRI scoring system, a prospectively evaluated cohort of consecutive cases of prenatally diagnosed isolated CCA that underwent fetal MRI and had known postnatal neurodevelopmental outcome assessed by a pediatric neurologist was selected. CCA was considered to be isolated when no other brain, spinal or extra‐CNS anomalies were detected on US or MRI antenatally and no chromosomal anomalies were identified, in accordance with previous publications^4^, ^18^, ^34^, ^35^, ^36^, ^37^, ^38^. The presence of an interhemispheric cyst or pericallosal lipoma was not an exclusion criterion^16^, ^17^, ^18^.

### Neurodevelopmental assessment

The Bayley Scales of Infant and Toddler Development, third edition (BSID‐III; German version)^39^ was used to estimate neurodevelopmental outcome in terms of gross and fine motor control, cognitive function and expressive and receptive language skills for children between the ages of 1 and 42.5 months. Normal development according to BSID‐III was defined as a development quotient (DQ) score of ≥ 85, and moderate‐to‐severe developmental delay was defined as a DQ score of < 70. Children over the age of 42.5 months were tested for cognitive and language skills using the Kaufman Assessment Battery for Children, second edition (KABC‐II; German version)^40^. Normal development according to KABC‐II was defined as a global scale index (GSI) of ≥ 85. As motor skill assessment is not included in KABC‐II, additional testing using the Peabody Developmental Motor Scales (0–5 years)^41^ or Bruininks–Oseretzky Test 2 (German version; 4–14 years)^42^ was applied depending on the age of the child. For the purpose of analysis of the correlation between fetal MRI score and neurodevelopmental outcome, individual neurodevelopmental tests for motor, language and cognitive skills were scored as follows: 0, below average (DQ or GSI < 85); 1, average (DQ or GSI 85–115); and 2, above average (DQ or GSI > 115). In each of the assessed neurodevelopmental fields, patients with average or above average development were considered to have a good outcome; patients with concomitant good cognitive and language development were considered to have an overall good outcome.

All children were included prospectively in a follow‐up program, with regular neurodevelopmental evaluations after birth and every 6 months thereafter, as well as neurophysical or movement therapy and occupational and speech therapy if needed.

### Imaging analysis

Images were scored independently by two neuroradiologists with experience in fetal MRI, blinded to all clinical information except gestational age (GA). When more than one examination was available, they were scored independently. The score was averaged between the two raters and rounded up to the nearest whole unit.

MRI examinations comprised CNS and fetal body assessment, in accordance with published guidelines^43^, performed at a tertiary center. For the fetal brain assessment, T2‐weighted single‐shot fast spin‐echo (ssFSE) in three orthogonal planes (slice thickness, 2–4 mm; slice gap, 0–0.4 mm; field of view, 230–260 mm, matrix 256), and T1‐weighted, diffusion‐weighted imaging (DWI) and echo‐planar imaging (EPI) in at least one orthogonal plane were acquired in all patients. Fetal body MRI evaluation comprised T2‐weighted steady‐state free precession sequences in three orthogonal planes, and T2‐weighted ssFSE, T1‐weighted, EPI and DWI images in at least one plane. No maternal anesthesia was administered. Further sequences were acquired depending on the examination findings and on fetal and maternal conditions.

### Statistical analysis

A mixed‐model intraclass correlation coefficient (ICC) for absolute agreement was used to assess inter‐rater agreement in fetal MRI score assessment. Spearman's rank correlation coefficient (ρ) was calculated to describe the correlation between fetal MRI score and neurodevelopmental score. Owing to the sample size, no multivariate regression models were calculated. In order to take into account multiple measurements per patient, mixed‐model ANOVA was used to compare MRI scores between cases assessed up to 24 + 6 weeks' gestation and cases assessed at or after 25 + 0 weeks; *P* < 0.05 was considered to indicate statistical significance. In order to avoid increasing the risk of type‐2 errors, no multiplicity corrections were performed. Analysis was performed using IBM SPSS Statistics for Windows version 25.0 (IBM Corp., Armonk, NY, USA).

## RESULTS

**Figure 1:**
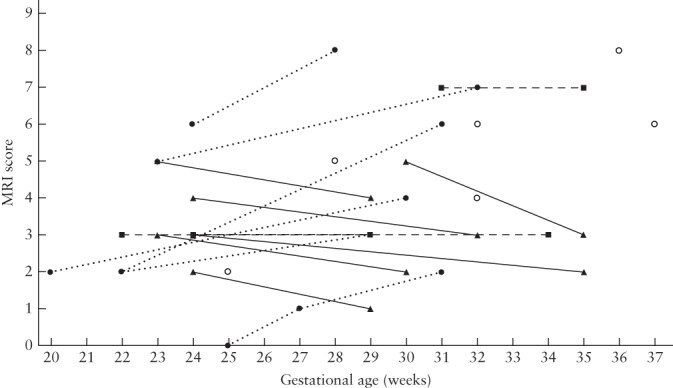
Magnetic resonance imaging (MRI) score in fetuses with isolated corpus callosal agenesis, according to gestational age. In fetuses with more than one examination, data points are interconnected, displaying evolution of score over time. Dotted lines represent cases in which score increased over time, solid lines represent cases in which score decreased and dashed lines represent cases in which score remained stable. Open circles are cases in which only one MRI examination was performed. Score is average of both raters, rounded up to closest unit.

Twenty‐one children fulfilled the inclusion criteria. Nine (42.9%) were female and 12 (57.1%) were male. A total of 37 fetal MRI examinations were evaluated in 20 pregnant women. Overall, mean GA at assessment was 28.3 ± 4.7 weeks (range, 20–38 weeks). Six patients had one fetal MRI examination during pregnancy (mean GA, 31.8 ± 4.8 weeks), 14 had two MRI examinations (mean GA at first scan, 26.5 ± 5.3 weeks; mean GA at second scan, 30.6 ± 2.4 weeks) and one patient had three MRI examinations performed (GA at first scan, 25 weeks; GA at second scan, 27 weeks; GA at third scan, 31 weeks) (Figure 1). There were two twin pregnancies; in one, which was monochorionic/monozygotic, both fetuses had CCA, while only twin 2 was affected in the other, which was dichorionic/dizygotic. Further characterization of the population can be found in Table 2. All children in our cohort were delivered after 35 weeks and there were no perinatal complications.

**Table 2:** Gestational age at magnetic resonance imaging (MRI), MRI score and neurodevelopmental (neurodev) outcome in 21 cases of fetal corpus callosal agenesis (CCA)

Case	Type of CCA	GA at first exam (weeks)	Fetal MRI score	Age at neurodev testing (years)	Neurodev outcome score*			Notes
					Cognitive	Motor	Language	
1	Partial (small body remnant)	21 + 1	1	1.7	1	1	1	
2	Partial (hypoplastic and dysplastic) + IH cyst	22 + 1	3	4.2	1	2	1	*DISP1* heterozygote
3	Complete	31 + 2	4	0.8	1	1	2	
4	Complete	24 + 3	2	2.9	1	0	2	
5	Partial (small body remnant)	23 + 3	3	3.1	1	2	1	
6	Partial (hypoplastic and dysplastic)	24 + 2	0	1.5	1	1	1	
7	Complete	22 + 4	4	3.5	0	0	0	
8	Partial (small body remnant)	23 + 0	2	2.0	1	1	1	*DCC* mutation
9	Partial (small body remnant)	19 + 1	2	3.8	1	1	1	
10	Partial + pericallosal lipoma	35 + 4	6	2.8	0	1	0	Negative WES
11	Partial (absent posterior body + splenium)	23 + 0	3	2.3	1	2	1	Negative WES
12	Complete + IH cyst	29 + 2	2	5.8	2	1	2	
13	Complete	22 + 1	4	2.0	0	0	1	Microdeletion Xq28
14	Complete	27 + 1	3	0.5	1	1	1	
15	Complete	21 + 4	2	0.6	1	1	2	
16	Partial (small body remnant)	23 + 0	4	2.2	0	0	0	*ARID1B* mutation
17	Complete	23 + 0	5	3.5	0	0	0	Severe delay; negative WES
18	Complete	21 + 4	2	0.6	1	1	2	
19	Complete	30 + 0	5	5.0	1	1	0	
20	Complete	31 + 0	3	1.4	1	1	1	
21	Partial (hypoplasia)	35 + 3	5	3.5	0	0	0	Severe delay

When more than one fetal MRI examination was performed, the first is presented.

* Neurodev outcome score: 0, below average (development quotient (DQ) or global scale index (GSI) < 85); 1, average (DQ/GSI 85–115); 2, above average (DQ/GSI > 115).

GA, gestational age; IH, interhemispheric; WES, whole‐exome sequencing.

Postnatal whole‐exome sequencing was available in seven patients. Mutations were detected in four of them: one with a mutation in the *ARID1B* gene (Coffin–Siris syndrome), one with a microdeletion (Xq28), one with a non‐relevant heterozygous mutation in the *DISP1* gene and one with a *DCC* mutation.

MRI scores ranged from 0 to 6 points, with a median of 3 points. Inter‐rater agreement was excellent (ICC, 0.959 (95% CI, 0.921–0.979)); most inconsistent ratings were related to opercularization (5/36). Variations in fetal MRI scores and timing of the examinations are shown in Figure 1. Six fetuses had an increase in MRI score between evaluations; this was at least partially due to an increase

in ventricular size in all cases, without modification of other characteristics in 5/6 cases.

**Figure 2:**
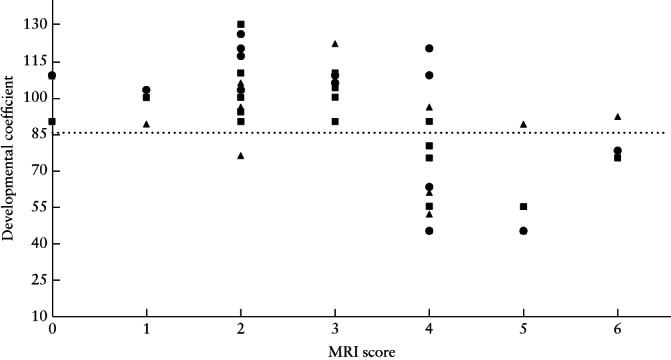
Neurodevelopmental outcome scores for each domain (cognitive (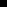), motor (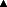) and language (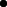) skills) in cases of isolated corpus callosal agenesis, according to fetal magnetic resonance imaging (MRI) score. Neurodevelopmental outcome was considered below average if developmental coefficient (DQ) or global scale index (GSI) was < 85 (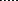), average if DQ/GSI was 85–115 and above average if DQ/GSI was > 115.

Neurodevelopmental evaluation was performed at an average age of 2.6 ± 1.46 years (range, 0.5–5.8 years).

Cognitive skills were within the average range (14/21) or above (1/21) in 15/21 (71.4%) children, motor skills were average (12/21) or above (3/21) in 15/21 (71.4%) children and language skills were average (10/21) or above (5/21) in 15/21 (71.4%) children (Table 2). Four children had poor scores in all developmental fields, and 28.6% (6/21) of the children had moderate‐to‐severe delay (Figure 2). Of seven patients with severe ventriculomegaly, four (57.1%) scored average or above in all three neurodevelopmental domains, with three (42.9%) patients having a poor outcome.

There was a significant negative correlation between fetal MRI score and neurodevelopmental outcome in the three fields tested: cognitive (ρ = –0.559, *P* < 0.0001), motor (ρ = –0.414, *P* = 0.012) and language (ρ = –0.565, *P* < 0.0001). There was a difference of 0.5 points in the mean fetal MRI score between children who had a

**Figure 3:**
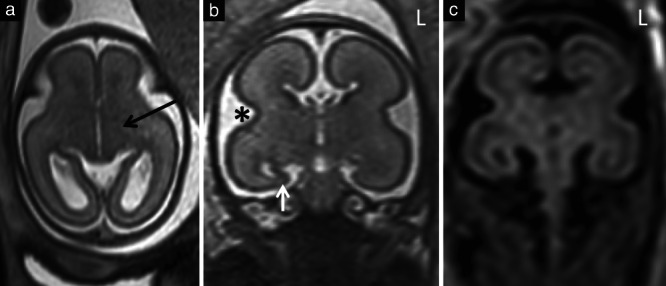
Example brain magnetic resonance images (MRI) in a fetus with isolated corpus callosal agenesis at 23 weeks' gestation that had a low MRI score. (a,b) T2‐weighted single‐shot fast‐spin echo images in axial (a) and coronal (b) planes. (c) T2‐weighted FLAIR image in coronal plane. There was no ventriculomegaly and normal basal ganglia (internal capsule (black arrow)) (a), normal opercularization () (b) and normal lamination, which was better identified on T2‐weighted FLAIR imaging (c). Features included unilateral mild hippocampal malrotation on the right (white arrow) (b), scoring 1 point, and inverted temporal lobe symmetry (b,c), with a ‘squarer’ temporal lobe in the coronal plane on the left (L), scoring 1 point.

**Figure 4:**
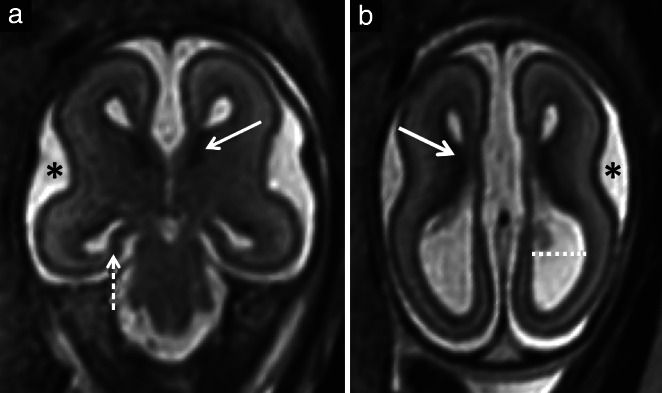
Example brain magnetic resonance images (MRI) in a fetus with isolated corpus callosal agenesis at 22 weeks' gestation that had a high MRI score. Coronal (a) and axial (b) T2‐weighted single shot fast‐spin echo images. There was delayed opercularization () (a,b), scoring 1 point, severely malrotated hippocampi that were almost completely flat (dashed arrow) (a), scoring 3 points, abnormal lamination with abnormally thick germinal matrix (solid arrows) (a,b), scoring 2 points, and moderate ventriculomegaly (dotted line) (b), scoring 1 point. (b) Unlike in Figure 3, lamination could be visualized adequately on T2‐weighted image.

MRI examination at or before 24 + 6 weeks and those who had a MRI examination at or after 25 weeks (2.6 *vs* 4.0 points). Children with a fetal MRI score of ≤ 3 points (13/21 children; 23/37 fetal MRI examinations) (Table 2, Figure 3) had average or above average neurodevelopmental outcome in all three fields, with the exception of one patient whose motor development was below average (with average cognitive development and above average language skills for age). Children with a fetal MRI score of ≥ 4 points (8/21 children; 14/37 fetal MRI examinations) (Table 2, Figure 4) scored below average on at least the cognitive or language evaluation (6/8 children scored below average in two or three domains), with the exception of one child with a fetal MRI score of four and normal neurodevelopment (cognitive score, 1; motor score, 1; language score, 2; age at testing, 9 months). Using the fetal MRI score cut‐offs of ≤ 3 points for good neurodevelopmental outcome and ≥ 4 points for high risk of poor neurodevelopmental outcome, the correct prognosis could be determined in 20/21 (95.2% (95% CI, 77.3–99.2%)) children and in 36/37 (97.3% (95% CI, 86.2%–99.5%)) fetal MRI examinations.

## DISCUSSION

Isolated CCA can be associated with neurodevelopmental outcomes ranging in severity from normal cognitive, motor and language skills to severe neurodevelopmental delay with full dependency on a carer for life. Relying on currently available clinical and imaging techniques, it is not possible to stratify accurately low‐ and high‐risk fetuses with isolated CCA. This is a pressing issue for prenatal counseling and a source of parental anxiety. The objective of the fetal MRI scoring system presented in this study is to facilitate patient counseling by clinicians when requested and to aid parental decision‐making.

By analyzing and scoring neuroanatomical anomalies in fetuses with otherwise isolated CCA, we propose a fetal MRI scoring system that improves risk stratification for these patients, showing a significant correlation with postnatal neurodevelopmental tests in three domains: motor, cognitive and language skills. The different components of the score were selected based on previous insights into the prenatal CCA phenotype^21^, ^22^.

It is difficult to determine what constitutes isolated CCA. There is no standard definition, with definitions varying between studies^35^, ^44^, but there is a certain degree of brain morphological change that is accepted in the context of CCA^4^. Most studies accept ventriculomegaly as part of the spectrum, with (≤ 15 mm^17^, ^45^ or ≤ 20 mm^16^) or without established limits^34^, ^36^, ^37^, ^44^, ^46^. Other anatomical features, such as temporal horn dilatation and hippocampal malrotation, are also considered on this spectrum^4^. Despite not constituting brain malformations, the accumulation of anatomical variations may point towards an underlying entity or have a cumulative effect on functional development. In our low‐risk group (fetal MRI score ≤ 3), no cases had abnormal findings on pre‐ or postnatal genetic testing.

We opted to validate the fetal MRI scoring system in a mixed cohort of isolated partial and complete CCA, since they have similar neurodevelopmental outcomes and present the same counseling challenges^15^, ^44^, ^47^, ^48^. Furthermore, we did not exclude a patient with callosal lipoma, as the outcome in such cases is determined by the associated malformations (isolated CCA in this case) and not on the lipoma itself^49^, ^50^.

Genetic anomalies detected postnatally were not taken into consideration, as these results were not available at the time of counseling. Extended prenatal genetic work‐up is becoming common in the treatment of CCA in many centers. However, it is not part of the standard patient care in many countries. These tests are invasive, time‐consuming and expensive, often not covered by healthcare plans and, depending on local laws, the results may not be available until after the legal limit for termination of pregnancy. The fetal MRI scoring system presented here is intended to serve as an easy‐to‐apply tool for risk stratification in cases of CCA. It does not replace other tests recommended when counseling CCA patients^51^, ^52^, and all information available should be integrated into this process. For similar reasons, we did not use information obtained from DTI or blood‐oxygenation‐level‐dependent imaging, as these techniques are not available everywhere and may not be feasible in all cases. It is, however, worth mentioning that characterization of fiber tracts and/or connectivity may help give important insights into this heterogeneous pathology.

There was a moderate negative correlation between neurodevelopmental outcome and fetal MRI score (ρ ≥ –0.414, *P* ≤ 0.012). A low fetal MRI score (≤ 3 points) was associated with average or above average early postnatal cognitive and language outcome in all cases (13/13 children), with one child scoring below average in motor skills who was still considered to have good neurodevelopmental outcome. Children with a fetal MRI score of ≥ 4 points had more variable outcomes, with one (1/8 children) scoring within the normal range in all three domains, three scoring within the normal range in one or two domains (one in language, one in motor and one in cognitive and motor) and four scoring below average in all three domains. If the proposed fetal MRI score with a cut‐off of 3 points is used, prognostication would be improved compared with that described previously, to accurate prognostication in 95.2% (20/21) of cases, particularly for cases with a good prognosis (13/13 cases).

In our cohort, 28.6% (6/21) of children had moderate‐to‐severe delay, which is in accordance with the rate in previously reported series^14^, ^15^, ^16^. Similarly to the study of Noguchi *et al*.^53^ and other reports^44^, this had no particular relationship with the degree of ventriculomegaly; of seven patients with severe ventriculomegaly, four (57.1%) scored average or above in all three neurodevelopmental domains, while three (42.9%) patients had a poor outcome. This may suggest that ventriculomegaly is not the defining factor of outcome.

There are several limitations to this study. The duration of follow‐up and the age at postnatal evaluation varied, and different testing, albeit standardized, had to be performed based on the age and abilities of the child. Furthermore, we do not have data on follow‐up into adulthood, when minor syndromes may manifest^9^. We would, however, argue that these cases would still be included in a ‘good‐neurological‐outcome’ group. Our cohort was relatively small and did not allow regression analysis for better fitting of the proposed fetal MRI score to outcome. A larger number of CCA cases are needed to ascertain if any particular feature relates to deficits in specific neurodevelopmental areas and to further stratify risk, particularly in the high‐scoring group. By publishing these initial findings, we hope that other research groups will be able to validate the data presented here in their cohorts. Application of this scoring system in clinical practice requires experience with fetal MRI. Such evaluation should be performed by an examiner experienced in the field; agreement of 96% is possible by experienced observers. So that this evaluation could be performed for all fetal MRI examinations, and even potentially applied to neurosonography, we did not at this time include advanced imaging techniques in the scoring system. Despite obvious advantages of fetal MRI in the assessment of cases of commissural agenesis^54^, neurosonography experts may be able to apply this score to US^55^, ^56^, ^57^. This would be an important future step towards a widely applicable tool that is independent of modality. However, it is important to emphasize that fetal brain development is an ongoing process, and more information, be it by advanced MRI techniques or by imaging fetuses at later GAs, may provide further ability to stratify risk using imaging in cases of CCA.

### Conclusions

By analyzing a variety of neuroanatomical features in fetuses with otherwise isolated CCA, we propose a new fetal MRI scoring system that improves the stratification of risk for severe neurodevelopmental delay. Further investigations, such as advanced genetic testing, should be performed, particularly in higher‐scoring cases.

## Supporting information

**AppendixS1** Detailed description of each parameter included in the fetal MRI scoring system for corpus callosal agenesis

**Figure S1** Brain magnetic resonance images in fetuses with isolated CCA, showing normal ventricular size (a) and mild/moderate (b,c) and severe (d) ventriculomegaly.
**Figure S2** Brain magnetic resonance images in a fetus with partial isolated CCA at 28 weeks, showing evaluation of lamination. Axial T2‐weighted ssFSE (a), DWI (b) and T2‐weighted FLAIR (c) images through the basal ganglia.
**Figure S3** Brain magnetic resonance images in a fetus with complete isolated CCA at 22 weeks, showing abnormal lamination with abnormally prominent and thick germinal matrix, at the level of the basal ganglia and above, scoring 2 points.
**Figure S4** Coronal T2‐weighted magnetic resonance image of the brain and body of a 29‐week fetus with complete isolated corpus callosal agenesis.
**Figure S5** Coronal T2‐weighted single shot magnetic resonance FSE sequences of the brain of the same fetus with complete isolated CCA at 24 weeks (a) and 28 weeks (b).
**Figure S6** Example magnetic resonance image of symmetrical temporal lobes in a 27‐week fetus with complete isolated CCA. Coronal T2‐weighted single shot FSE sequence.
**Figure S7** Grading of hippocampus position on magnetic resonance imaging in fetuses with isolated CCA. Coronal T2‐weighted ssFSE images at 24 (a), 31 (b), 23 (c) and 28 (d) weeks in fetuses with complete (a,c,d) or partial (b) CCA.
**Figure S8** Axial T2‐weighted magnetic resonance ssFSE images of fetus with complete isolated CCA at 28 weeks (a) and of fetus with partial isolated CCA at 30 weeks (b).
**Figure S9** Side‐by‐side comparison of lamination on magnetic resonance imaging of a normal fetal brain at 24 weeks. Axial T2‐weighted images (a,e), DWI Zoom (b,f), DWI (c,g), and T2‐weighted FLAIR (d,h).
