# Supplementary material for: Improved neurodevelopmental prognostication in isolated corpus callosal agenesis: fetal magnetic resonance imaging‐based scoring system
Source: Ultrasound Obstet Gynecol. 2021 Jul 1;58(1):34–41. doi: 10.1002/uog.22102 (PMC8362015; doi:10.1002/uog.22102)
Supplement: Supplementary file 1 — AppendixS1 Detailed description of each parameter included in the fetal MRI scoring system for corpus callosal agenesis [file UOG-58-34-s001.docx]

**Appendix S1** Detailed description of each parameter included in the fetal MRI scoring system for corpus callosal agenesis

**Introduction**

The aim of this document is to facilitate standardization and application of the proposed fetal MRI score presented in the paper entitled “Improved prognostication in isolated callosal agenesis: a fetal magnetic resonance imaging-based scoring system”.

It encloses a resumed explanation of the application of this novel MRI scoring system in corpus callosum agenesis (CCA). Further, it aims to provide a summarized insight into the rationale for each imaging parameter choice and supply the readers with the adequate literature references.

These will be presented in a category-wise approach.

**I - VENTRICULOMEGALY/PARENCHYMAL LOSS**

Colpocephaly is a known consequence of corpus callosum agenesis (CCA). This is often associated with ventriculomegaly, particularly of the posterior aspect of lateral ventricles, ranging from normal sized ventricles to a severe ventricular dilatation. The etiology and clinical significance of this enlargement remains a topic of discussion, but it is mostly assumed to be part of the disease spectrum.^1-3^ Mild to moderate ventriculomegaly in fetuses without other associated anomalies is most often related with a good neurodevelopment outcome, while severe ventriculomegaly has per se a higher risk of developmental delay and neurologic dysfunction.^4^

Although we initially planned to use “parenchymal loss” as the scoring parameter, we found this correlated well with the previously established degrees of ventriculomegaly.^4,5^ Therefore, we chose 2D ventricular width as a surrogate measurement, which is easily and reliably assessed on MRI and US, in full appreciation that this parameter may be replaced by more detailed 3D measurements in the future.

As previously described, ventricle width should be measured on an axial image that passes through the thalami, at the posterior margin of the glomus of the choroid plexus.^5^ All measurements should be based on coronal T2-weighted (T2w) sequences, as they are most comparable to standardized neurosonographic measurements.^6^ If the ventricles are asymmetrical, the larger ventricle width should be used. Normal atrial size was considered when the atria had a width of <10mm, mild (score 1) 10-15mm and severe >15mm (score 2).

**II - SULCATION**

The study of sulcation and gyration is a paramount step in fetal brain assessment and an indisputable contribution of fetal MR. The different sulci (primary, secondary and tertiary) appear in a precise order and timing. They allow a reliable estimate of the gestational age of the fetus and are an excellent marker of fetal brain maturation.^7-9^ There is however some variability in the detection of sulci and gyri. Usually, a mean lag time of two-week between the detectability of a sulcus and its presence in 75% or more of the cases can be expected.^9^ A detected delay under two GW is often considered within normal and close follow up should be performed to assure no further anomalies are detected and gyration progresses adequately. The CC develops in a period while cortical folding is starting and as such could be expected to influence this process amply. However, MRI studies have shown no statistically significant difference from normal individuals, despite a slight trend for CCA fetuses to score younger.^10^

Having all of this is mind, we opted to divide this category into 3 levels: “normal” (score of 0), a score of 1, if the delay is mild, meaning less than 2 weeks difference from the normal, and “delayed" if this delay is superior to 2 GW, scoring 2 points, as this may point towards a severe concurrent underlying issue.

For this assessment, use of know atlases may be helpful.^10-14^

**III - LAMINATION**

Beyond evaluation of sulcation and gyration patterns, a paramount part of fetal brain appraisal is the assessment of telencephalic lamination. As the fetal brain develops, several permanent and transient layers can be identified, in a time specific pattern.^15,16^ These represent a macroscopic translation of microscopic processes of brain cell differentiation, migration, and organization. Further, some brain malformations, such as lissencephalies, only show their full morphological impact in later pregnancy. At earlier stages, their only sign is an abnormal appearance of fetal brain lamination.

Identification of the cortical plate and germinal matrix is straightforward on T2WI, evaluation of the subplate and intermediate zone (IZ) is limited after 24-26 GW^17,18^, particularly if image quality or signal-to-noise ratio are not ideal. Other sequences may be used depending on availability including T2-w FLAIR images^19^ and DWI.^20^ The subplate can be identified on T2-w FLAIR images until birth, initially as a continuous hypointense subcortical band, and progressing with gyration disappearing initially at the bottom of the sulci, but remaining at the gyral crests.^19^ Roughly, the germinal matrix should start to involute at 26 GW and not be seen by 28-29 GW on T2WI.^16,18^ Attention should be paid particularly to the “crossroad” regions (periventricular triangles),^21,50^ which are often affected in CCA cases, as well as persistence or abnormal thickness of the germinal matrix, which may represent arrested/abnormal migration of neurons and may be the only precocious sign of a migrational disorder.

Lamination evaluation was binary into “normal” or “abnormal”, with a weight of 2 for abnormal lamination, given its severity in terms of development.

**IV - OPERCULARIZATION**

Opercularization refers to the progressive covering of the insular cortex by the frontal, temporal, and parietal opercula. The insula begins to invaginate from the cerebral surface at 14-16GW. At 20-22GW, opercularization initiates and proceeds in a well-defined and predetermined manner, until at full term, the opercula completely cover the insula.^22,23^ The opercula and insula comprise areas important for language, auditory, and secondary somatic sensory and motor functions, and developmental anomalies of this area may cause significant impairment with developmental delays.^22-25^ Examples of abnormal opercularization include metabolic disorders and other genetic syndromes (i.e. Soto syndrome).

Most fetal neuroimaging experts have their subjective yet reliable ways in assessing opercularization. Using fetal MRI, it is most often assessed in the coronal plane (perpendicular to the fetal brainstem).^8,11,13^ In fetal neurosonography the assessment in the axial plane is more standardized.^25^

Opercularization delay/anomaly may be seen in CCA, particular when in the context of a central nervous system-associated syndrome.^1,23^ As such we included this feature in the score, in a 0-2 scoring. Delayed opercularization (Score 1) would comprise any relative shallow depth of the insular invagination or wider opercular opening than expected for the gestational age. Abnormal opercularization refers to any irregular pattern of opercularization such as increased or misguided formation of one (or more) of the opercula or any other anomalies short of a specific malformation (in isolated CCA).

**V - TEMPORAL ASYMMETRY**

Structural asymmetry of the fetal brain is determined early in human development and has long been known from postmortem studies.^26^ This appears to be related to asymmetric hemispheric expression of genes gene, as early as 12 GW.^27^ Asymmetrical development of the temporal lobes (TL) has been described in normal fetuses by fetal MRI.^28^ In normal development, there is an earlier appearance of the right superior temporal sulcus (STS) at 23 GW (25 GW on the left side). The right STS is also deeper than its left counterpart in over 90% of cases.^28^ These structural asymmetries are associated with functional asymmetries.^29,30^ They involve language-related regions (*planum temporale*, STS) and symmetric development or inverted symmetry of the temporal lobes has been associated with abnormal language representation^31^ and later life with developmental language disorders and autism spectrum disorders.^32,33^ In callosal agenesis, these structural asymmetries are further influenced by development of and selective pruning of callosal fibers or lack-thereof,^29^ and this may be a translation of a genetic determined syndrome or epigenetic modulation.^32^ There are still several uncertainties regarding the clinical impact of altered symmetry (inverted or symmetrical TL). However, abnormal symmetry patterns in fetuses with CCA are occasionally found and indicate a specific deviation from the normal course of development, as they are rarely found in the normal population, we decided to include it in the scoring system, as it adds another discriminating feature in so called *isolated* CCA cases.

We opted to score normal TL asymmetry as follows: Normal TL asymmetry scores 0 points and should take into consideration the (con)formation of the *planum temporale* and the STS. The appearance of the TL contours should show significant asymmetry by 20GW, with a more “squared” appearance on the right and rounder appearance of the *planum* on the left. The STS should be visible at 23GW on the right and appears about 10 days earlier than on the left side. Care should be taken when analyzing this to make sure body structures can be clearly visible to determine lateralization (p. ex. Stomach on the left, liver right) and make sure to be in presence of a *situs solitus*. Symmetrical or inverted/abnormal development of the temporal lobes should be scored as “1”.

**VI - HIPPOCAMPAL POSITION**

The hippocampus is a paramount structure for cognitive function, learning and memory.^34^ During gestation, the hippocampus increases progressively in size and gradually rotates upwards to achieve its adult position.^3,35,36^ Abnormal rotation of the hippocampus has been connected to numerous pathological processes including epilepsy^37^, autism spectrum disorders^38^ and schizophrenia, as well as CCA^39^, to name a few. ^40^

In CCA hippocampus have been established to be significantly smaller than normals, and this difference was more notorious in associated than isolated CCA.^41^ The specific impact the hippocampal underdevelopment in CCA will have on postnatal cognitive function, learning and memory-processing domains is, however, yet to be assessed.

To determine normal hippocampal rotation, it is useful to use atlases (as described above) and/or angles, if there are any doubts see Rhigini et al 2006^36^. We divided these anomalies in two grades: minor malrotation that may be uni- or bilateral (please pay attention to the fact that there is a left-right asymmetry described in this, with the right hippocampus rotating sooner^40,42^) or unilateral clear verticalization. A bilaterally verticalized hippocampus (this should be evident even to a not so trained eye) or if there is associated atrophy, should score 2 points.

**VII - BASAL GANGLIA**

The basal ganglia (BG) are paramount in multiple cerebral functions including movement control, learning, cognition, and behaviour. ^43,44^

Although their evaluation prenatally is somewhat limited, it is essential in determination of normal brain development, and lesions to this region have been well documented on fetal MRI and abnormal volumes have been correlated to (worse) neurodevelopment outcome.^45,46^ Abnormal BG in association with CCA or dysplastic CC may be identified for instance in tubulinopathies.^47,48^ Although malformations of this region would not constitute “isolated CCA”, including this aspect in the score had a two-fold intention: “forcing” the ruling out of malformations of this region and hence confirming the isolated nature of the CCA, and detecting minor anomalies such as volume variance and inability to identify the internal capsule. Findings were classified in a bimodal fashion in “Normal” or “Abnormal”, with abnormal comprising all of the above mentioned.

Evaluation of the basal ganglia was performed on a visual basis using T2WI to assess signal intensity and volume, as well as DWI/DTI native images to better visualize the internal capsule.

**Technical considerations**

To improve diagnostic accuracy of fetal MRI (or any other imaging techniques) it is important to: 1) have knowledge and experience with fetal brain imaging, particularly MRI, development and pathology, 2) apply a correct MRI protocol.

Although there is no specific training in fetal MRI imaging, medical professionals should have sufficient experience when reading these examinations, as to be able to detect/rule out associated anomalies, but also be familiar with normal development to be able to identify subtle delays/anomalies.

Protocols for fetal MRI have been discussed, and broad guidelines exist.^49^ For evaluation of the fetal CNS, T2-w single shot images should be acquired in all three orthogonal planes, in relation to the brainstem. In addition, T1WI, a blood sensitive sequence (echo planar imaging, T2*, susceptibility-weighted imaging) and a diffusion-weighted sequence must be acquired in at least one plane. For evaluation of lamination we would further suggest the use of T2-w FLAIR images in the axial and coronal plane, or alternatively DWI/zoom diffusion also in two planes. There are also two main schools of thoughts: one defending brain imaging alone, or always imaging the whole fetal body, independently of indication. Reasonings behind each are beyond the scope of the present manuscript. However, at least one sequence covering the fetal body in the coronal plane should be available for lateralization of CNS structures. If a detailed ultrasound is not available, MRI may also be essential in excluding extra-CNS anomalies.

**References**

1. Glenn OA, Goldstein RB, Li KC, et al. Fetal magnetic resonance imaging in the evaluation of fetuses referred for sonographically suspected abnormalities of the corpus callosum. *J Ultrasound Med.* 2005;24(6):791-804.

2. Masmejan S, Blaser S, Keunen J, et al. Natural History of Ventriculomegaly in Fetal Agenesis of the Corpus Callosum. *J Ultrasound Med.* 2019.

3. Baker LL, Barkovich AJ. The large temporal horn: MR analysis in developmental brain anomalies versus hydrocephalus. *AJNR Am J Neuroradiol.* 1992;13(1):115-122.

4. Gaglioti P, Danelon D, Bontempo S, Mombro M, Cardaropoli S, Todros T. Fetal cerebral ventriculomegaly: outcome in 176 cases. *Ultrasound Obstet Gynecol.* 2005;25(4):372-377.

5. Cardoza JD, Goldstein RB, Filly RA. Exclusion of fetal ventriculomegaly with a single measurement: the width of the lateral ventricular atrium. *Radiology.* 1988;169(3):711-714.

6. Garel C, Alberti C. Coronal measurement of the fetal lateral ventricles: comparison between ultrasonography and magnetic resonance imaging. *Ultrasound Obstet Gynecol.* 2006;27(1):23-27.

7. Glenn OA, Barkovich AJ. Magnetic resonance imaging of the fetal brain and spine: an increasingly important tool in prenatal diagnosis, part 1. *AJNR Am J Neuroradiol.* 2006;27(8):1604-1611.

8. Garel C, Chantrel E, Elmaleh M, Brisse H, Sebag G. Fetal MRI: normal gestational landmarks for cerebral biometry, gyration and myelination. *Childs Nerv Syst.* 2003;19(7-8):422-425.

9. Garel C, Chantrel E, Brisse H, et al. Fetal cerebral cortex: normal gestational landmarks identified using prenatal MR imaging. *AJNR Am J Neuroradiol.* 2001;22(1):184-189.

10. Warren DJ, Connolly DJA, Griffiths PD. Assessment of Sulcation of the Fetal Brain in Cases of Isolated Agenesis of the Corpus Callosum Using In Utero MR Imaging. *American Journal of Neuroradiology.* 2010;31(6):1085-1090.

11. Levine D. *Atlas of Fetal MRI.* Vol 1: CRC Press; 2005.

12. Prayer D. *Fetal MRI.* 1 ed: Springer; 2011.

13. Griffiths P. *Atlas of fetal and postnatal brain MR.* Philadelphia, PA: Mosby/Elsevier; 2010.

14. Chapman T, Matesan M, Weinberger E, Bulas DI. Digital atlas of fetal brain MRI. *Pediatr Radiol.* 2010;40(2):153-162.

15. Bystron I, Blakemore C, Rakic P. Development of the human cerebral cortex: Boulder Committee revisited. *Nat Rev Neurosci.* 2008;9(2):110-122.

16. Kostovic I, Jovanov-Milosevic N, Rados M, et al. Perinatal and early postnatal reorganization of the subplate and related cellular compartments in the human cerebral wall as revealed by histological and MRI approaches. *Brain Struct Funct.* 2014;219(1):231-253.

17. Perkins L, Hughes E, Srinivasan L, et al. Exploring cortical subplate evolution using magnetic resonance imaging of the fetal brain. *Dev Neurosci.* 2008;30(1-3):211-220.

18. Widjaja E, Geibprasert S, Mahmoodabadi SZ, Blaser S, Brown NE, Shannon P. Alteration of human fetal subplate layer and intermediate zone during normal development on MR and diffusion tensor imaging. *AJNR Am J Neuroradiol.* 2010;31(6):1091-1099.

19. Diogo MC, Prayer D, Gruber GM, et al. Echo-planar FLAIR Sequence Improves Subplate Visualization in Fetal MRI of the Brain. *Radiology.* 2019;292(1):159-169.

20. Kasprian G, Del Rio M, Prayer D. Fetal diffusion imaging: pearls and solutions. *Top Magn Reson Imaging.* 2010;21(6):387-394.

21. Judas M, Rados M, Jovanov-Milosevic N, Hrabac P, Stern-Padovan R, Kostovic I. Structural, immunocytochemical, and mr imaging properties of periventricular crossroads of growing cortical pathways in preterm infants. *AJNR Am J Neuroradiol.* 2005;26(10):2671-2684.

22. Afif A, Bouvier R, Buenerd A, Trouillas J, Mertens P. Development of the human fetal insular cortex: study of the gyration from 13 to 28 gestational weeks. *Brain Struct Funct.* 2007;212(3-4):335-346.

23. Chen CY, Zimmerman RA, Faro S, et al. MR of the cerebral operculum: abnormal opercular formation in infants and children. *AJNR Am J Neuroradiol.* 1996;17(7):1303-1311.

24. Tatum WO, Coker SB, Ghobrial M, Abd-Allah S. The open opercular sign: diagnosis and significance. *Ann Neurol.* 1989;25(2):196-199.

25. Quarello E, Stirnemann J, Ville Y, Guibaud L. Assessment of fetal Sylvian fissure operculization between 22 and 32 weeks: a subjective approach. *Ultrasound Obstet Gynecol.* 2008;32(1):44-49.

26. Shapleske J, Rossell SL, Woodruff PW, David AS. The planum temporale: a systematic, quantitative review of its structural, functional and clinical significance. *Brain Res Brain Res Rev.* 1999;29(1):26-49.

27. Sun T, Patoine C, Abu-Khalil A, et al. Early asymmetry of gene transcription in embryonic human left and right cerebral cortex. *Science.* 2005;308(5729):1794-1798.

28. Kasprian G, Langs G, Brugger PC, et al. The prenatal origin of hemispheric asymmetry: an in utero neuroimaging study. *Cereb Cortex.* 2011;21(5):1076-1083.

29. Benezit A, Hertz-Pannier L, Dehaene-Lambertz G, et al. Organising white matter in a brain without corpus callosum fibres. *Cortex.* 2015;63:155-171.

30. Cykowski MD, Kochunov PV, Ingham RJ, et al. Perisylvian sulcal morphology and cerebral asymmetry patterns in adults who stutter. *Cereb Cortex.* 2008;18(3):571-583.

31. Schuler AL, Bartha-Doering L, Jakab A, et al. Tracing the structural origins of atypical language representation: consequences of prenatal mirror-imaged brain asymmetries in a dizygotic twin couple. *Brain Struct Funct.* 2018;223(8):3757-3767.

32. Herbert MR. Brain asymmetries in autism and developmental language disorder: a nested whole-brain analysis. *Brain.* 2004;128(1):213-226.

33. Leonard CM, Eckert MA. Asymmetry and dyslexia. *Dev Neuropsychol.* 2008;33(6):663-681.

34. Bohbot VD, Allen JJ, Nadel L. Memory deficits characterized by patterns of lesions to the hippocampus and parahippocampal cortex. *Ann N Y Acad Sci.* 2000;911:355-368.

35. Jacob FD, Habas PA, Kim K, et al. Fetal hippocampal development: analysis by magnetic resonance imaging volumetry. *Pediatr Res.* 2011;69(5 Pt 1):425-429.

36. Righini A, Zirpoli S, Parazzini C, et al. Hippocampal infolding angle changes during brain development assessed by prenatal MR imaging. *AJNR Am J Neuroradiol.* 2006;27(10):2093-2097.

37. Bajic D, Kumlien E, Mattsson P, Lundberg S, Wang C, Raininko R. Incomplete hippocampal inversion-is there a relation to epilepsy? *Eur Radiol.* 2009;19(10):2544-2550.

38. Campbell LE, Daly E, Toal F, et al. Brain and behaviour in children with 22q11.2 deletion syndrome: a volumetric and voxel-based morphometry MRI study. *Brain.* 2006;129(Pt 5):1218-1228.

39. Atlas SW, Zimmerman RA, Bilaniuk LT, et al. Corpus callosum and limbic system: neuroanatomic MR evaluation of developmental anomalies. *Radiology.* 1986;160(2):355-362.

40. Cury C, Toro R, Cohen F, et al. Incomplete Hippocampal Inversion: A Comprehensive MRI Study of Over 2000 Subjects. *Front Neuroanat.* 2015;9:160.

41. Knezovic V, Kasprian G, Stajduhar A, et al. Underdevelopment of the Human Hippocampus in Callosal Agenesis: An In Vivo Fetal MRI Study. *AJNR Am J Neuroradiol.* 2019;40(3):576-581.

42. Bajic D, Canto Moreira N, Wikstrom J, Raininko R. Asymmetric development of the hippocampal region is common: a fetal MR imaging study. *AJNR Am J Neuroradiol.* 2012;33(3):513-518.

43. Herrero MT, Barcia C, Navarro JM. Functional anatomy of thalamus and basal ganglia. *Childs Nerv Syst.* 2002;18(8):386-404.

44. Baez-Mendoza R, Schultz W. The role of the striatum in social behavior. *Front Neurosci.* 2013;7:233.

45. Girard N, Confort-Gouny S, Schneider J, et al. MR imaging of brain maturation. *J Neuroradiol.* 2007;34(5):290-310.

46. Sanz-Cortes M, Ratta GA, Figueras F, et al. Automatic quantitative MRI texture analysis in small-for-gestational-age fetuses discriminates abnormal neonatal neurobehavior. *PLoS One.* 2013;8(7):e69595.

47. Bahi-Buisson N, Poirier K, Fourniol F, et al. The wide spectrum of tubulinopathies: what are the key features for the diagnosis? *Brain.* 2014;137(Pt 6):1676-1700.

48. Brock S, Stouffs K, Scalais E, et al. Tubulinopathies continued: refining the phenotypic spectrum associated with variants in TUBG1. *Eur J Hum Genet.* 2018;26(8):1132-1142.

49. Prayer D, Malinger G, Brugger PC, et al. ISUOG Practice Guidelines: performance of fetal magnetic resonance imaging. *Ultrasound Obstet Gynecol.* 2017;49(5):671-680.

50. Milos RI, Jovanov-Milošević N, Mitter C, Bobić-Rasonja M, Pogledic I, Gruber GM, Kasprian G, Brugger PC, Weber M, Judaš M, Prayer D. Developmental dynamics of the periventricular parietal crossroads of growing cortical pathways in the fetal brain - In vivo fetal MRI with histological correlation. *Neuroimage*. 2020;15;210:116553. doi: 10.1016/j.neuroimage.2020.116553.
