# Supplementary material for: Improved neurodevelopmental prognostication in isolated corpus callosal agenesis: fetal magnetic resonance imaging‐based scoring system
Source: Ultrasound Obstet Gynecol. 2021 Jul 1;58(1):34–41. doi: 10.1002/uog.22102 (PMC8362015; doi:10.1002/uog.22102)
Supplement: Supplementary file 2 — Figure S1 Brain magnetic resonance images in fetuses with isolated CCA, showing normal ventricular size (a) and mild/moderate (b,c) and severe (d) ventriculomegaly. Figure S2 Brain magnetic resonance images in a fetus with partial isolated CCA at 28 weeks, showing evaluation of lamination. Axial T2‐weighted ssFSE (a), DWI (b) and T2‐weighted FLAIR (c) images through the basal ganglia. Figure S3 Brain magnetic resonance images in a fetus with complete isolated CCA at 22 weeks, showing abnormal lamination with abnormally prominent and thick germinal matrix, at the level of the basal ganglia and above, scoring 2 points. Figure S4 Coronal T2‐weighted magnetic resonance image of the brain and body of a 29‐week fetus with complete isolated corpus callosal agenesis. Figure S5 Coronal T2‐weighted single shot magnetic resonance FSE sequences of the brain of the same fetus with complete isolated CCA at 24 weeks (a) and 28 weeks (b). Figure S6 Example magnetic resonance image of symmetrical temporal lobes in a 27‐week fetus with complete isolated CCA. Coronal T2‐weighted single shot FSE sequence. Figure S7 Grading of hippocampus position on magnetic resonance imaging in fetuses with isolated CCA. Coronal T2‐weighted ssFSE images at 24 (a), 31 (b), 23 (c) and 28 (d) weeks in fetuses with complete (a,c,d) or partial (b) CCA. Figure S8 Axial T2‐weighted magnetic resonance ssFSE images of fetus with complete isolated CCA at 28 weeks (a) and of fetus with partial isolated CCA at 30 weeks (b). Figure S9 Side‐by‐side comparison of lamination on magnetic resonance imaging of a normal fetal brain at 24 weeks. Axial T2‐weighted images (a,e), DWI Zoom (b,f), DWI (c,g), and T2‐weighted FLAIR (d,h). [file UOG-58-34-s002.docx]

**Supplementary Figures**


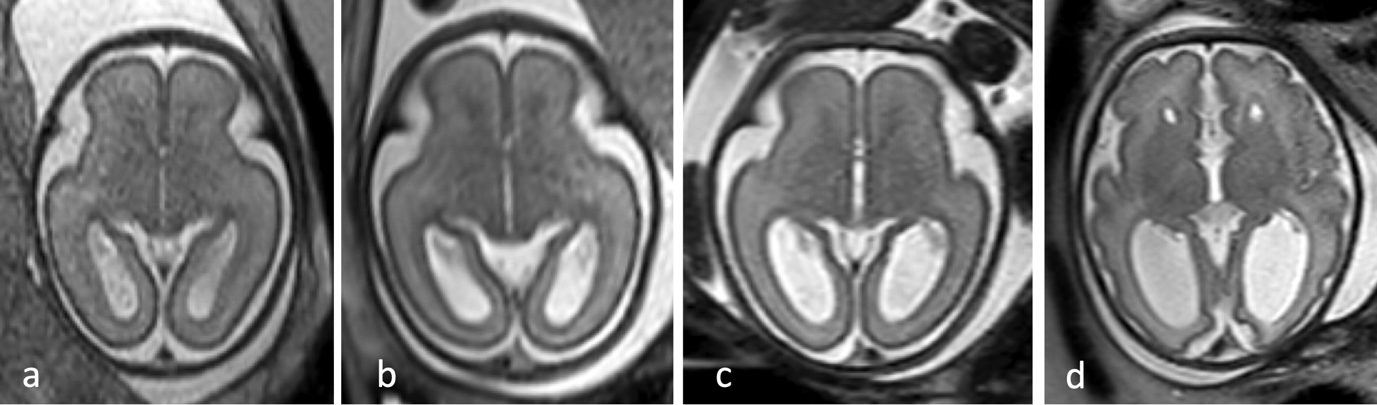


**Figure S1**


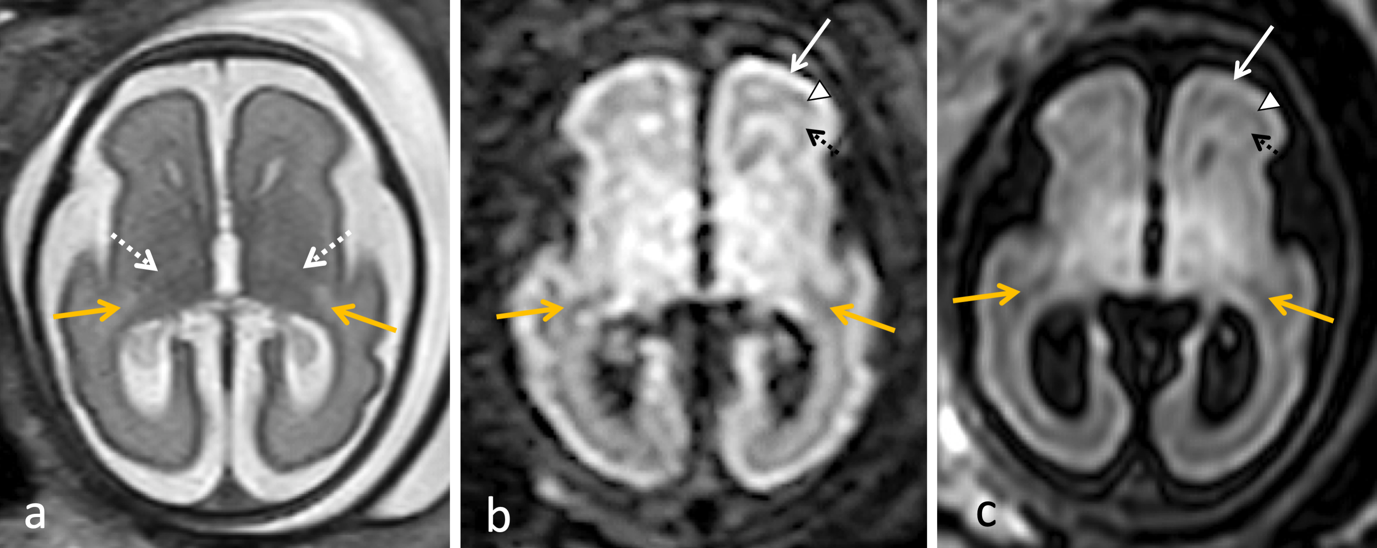


**Figure S2**


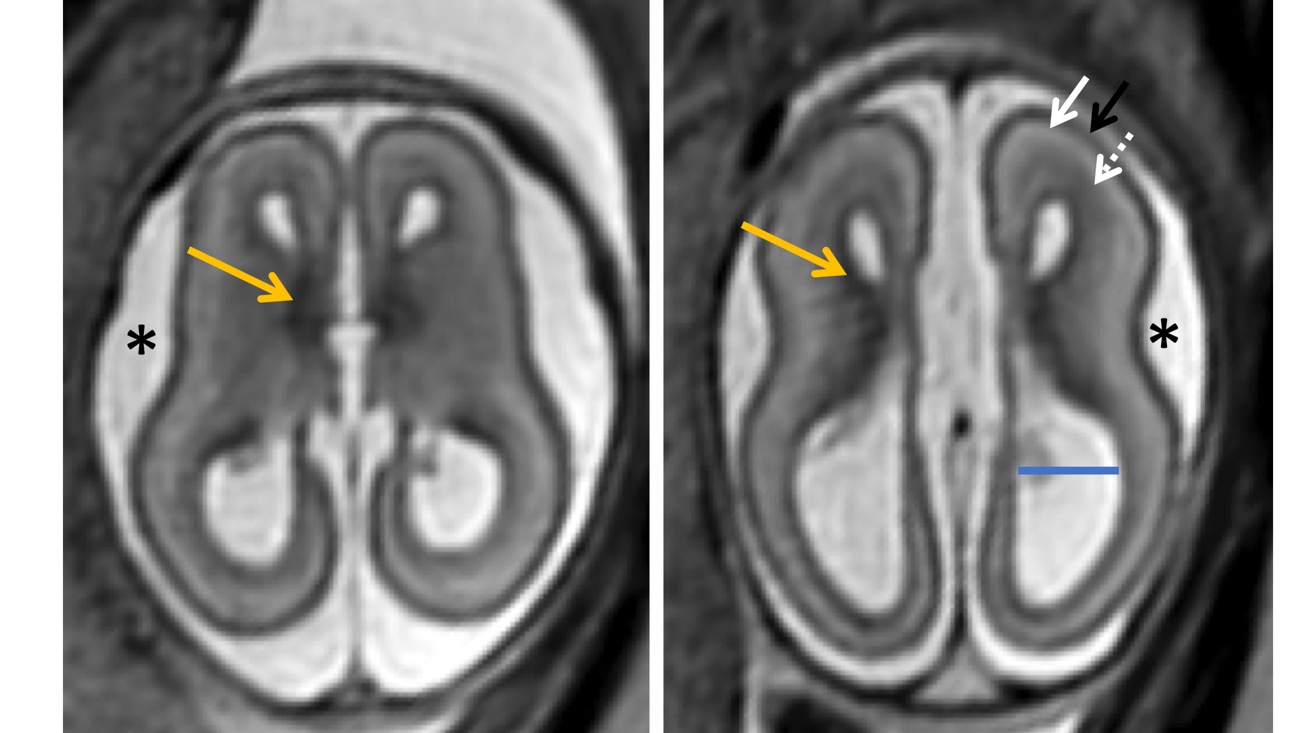


**Figure S3**


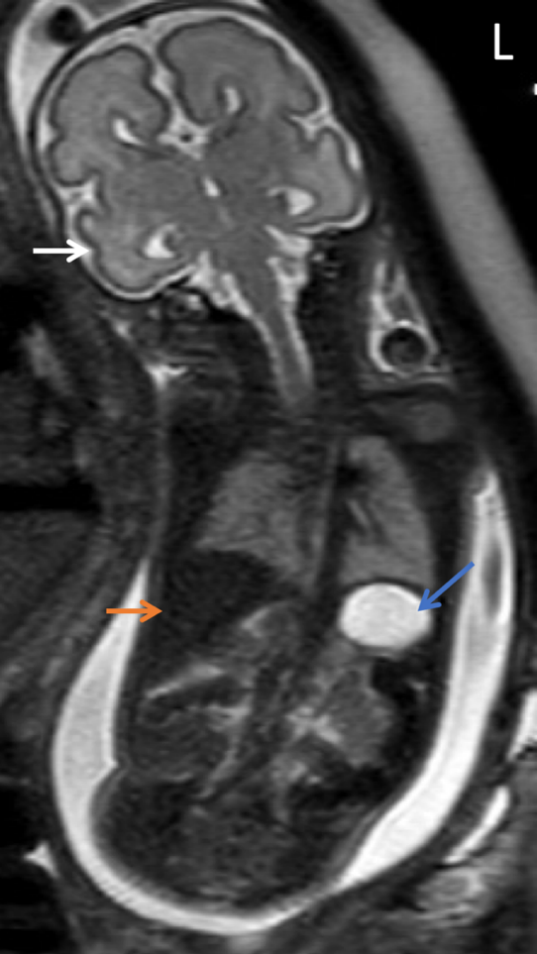


**Figure S4**


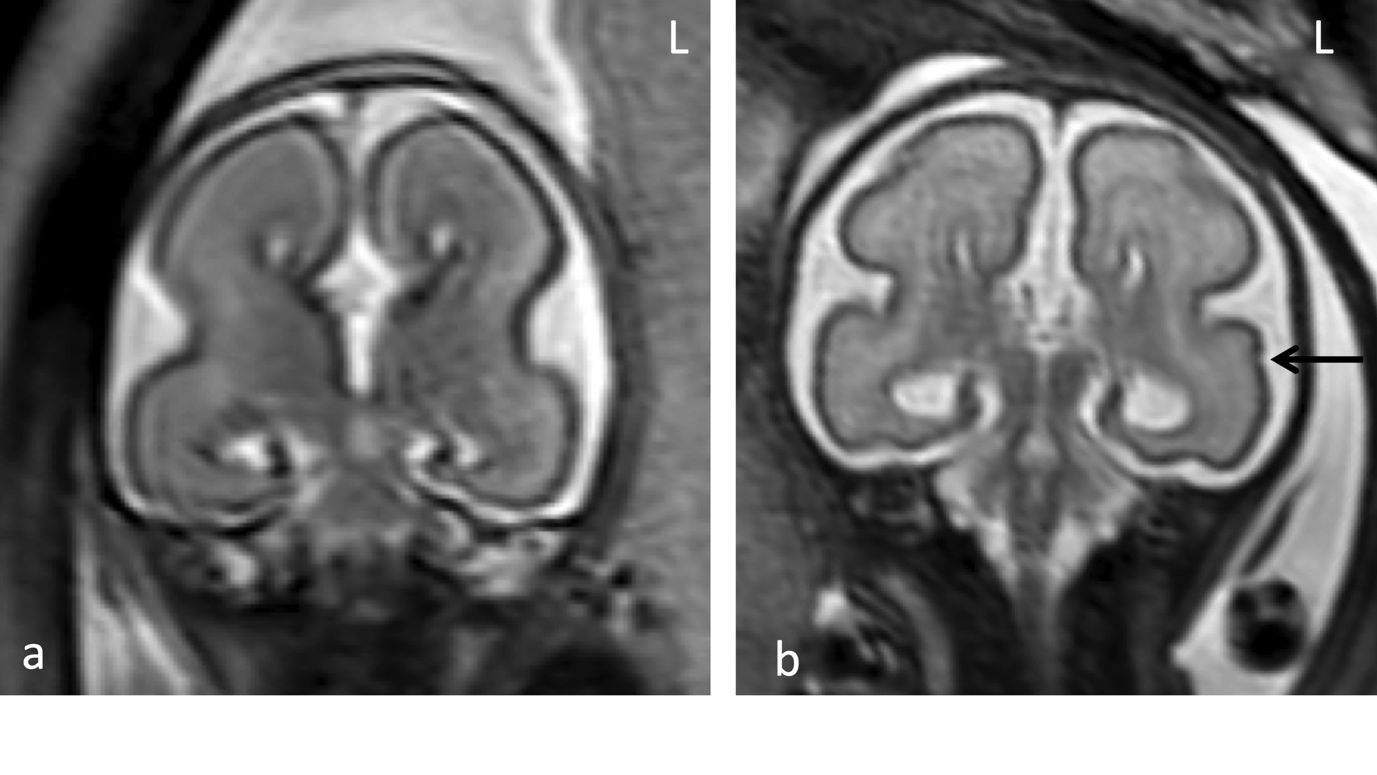


**Figure S5**


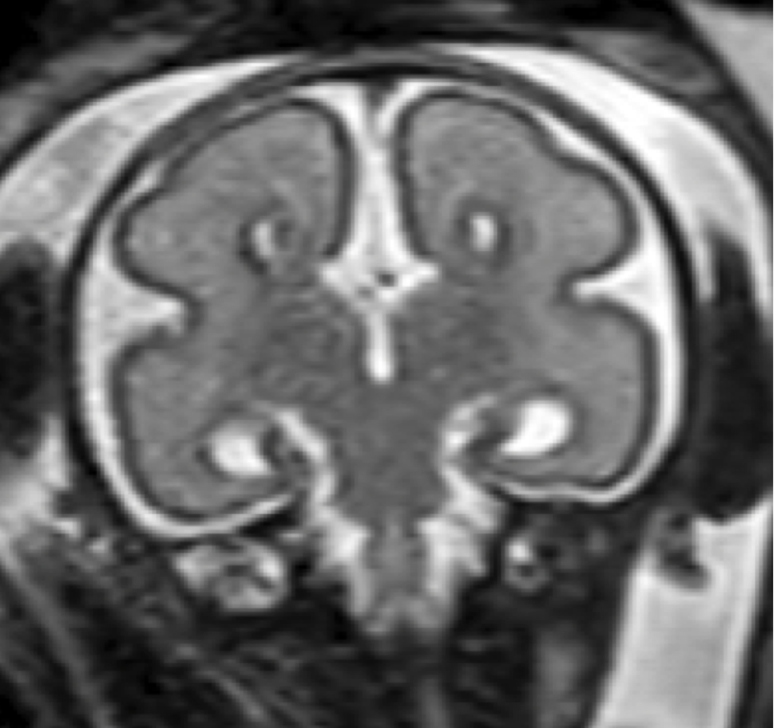


**Figure S6**


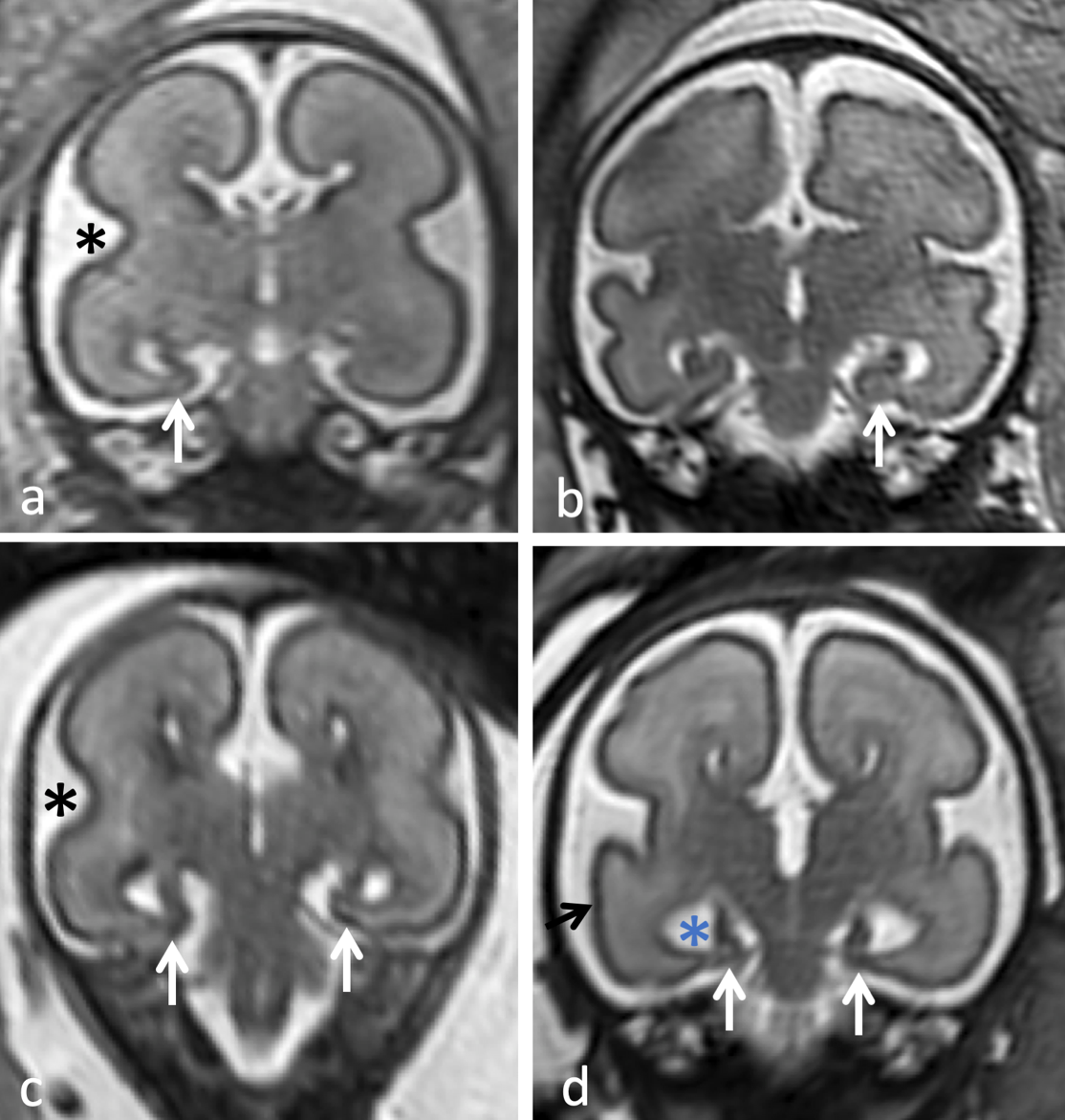


**Figure S7**

**
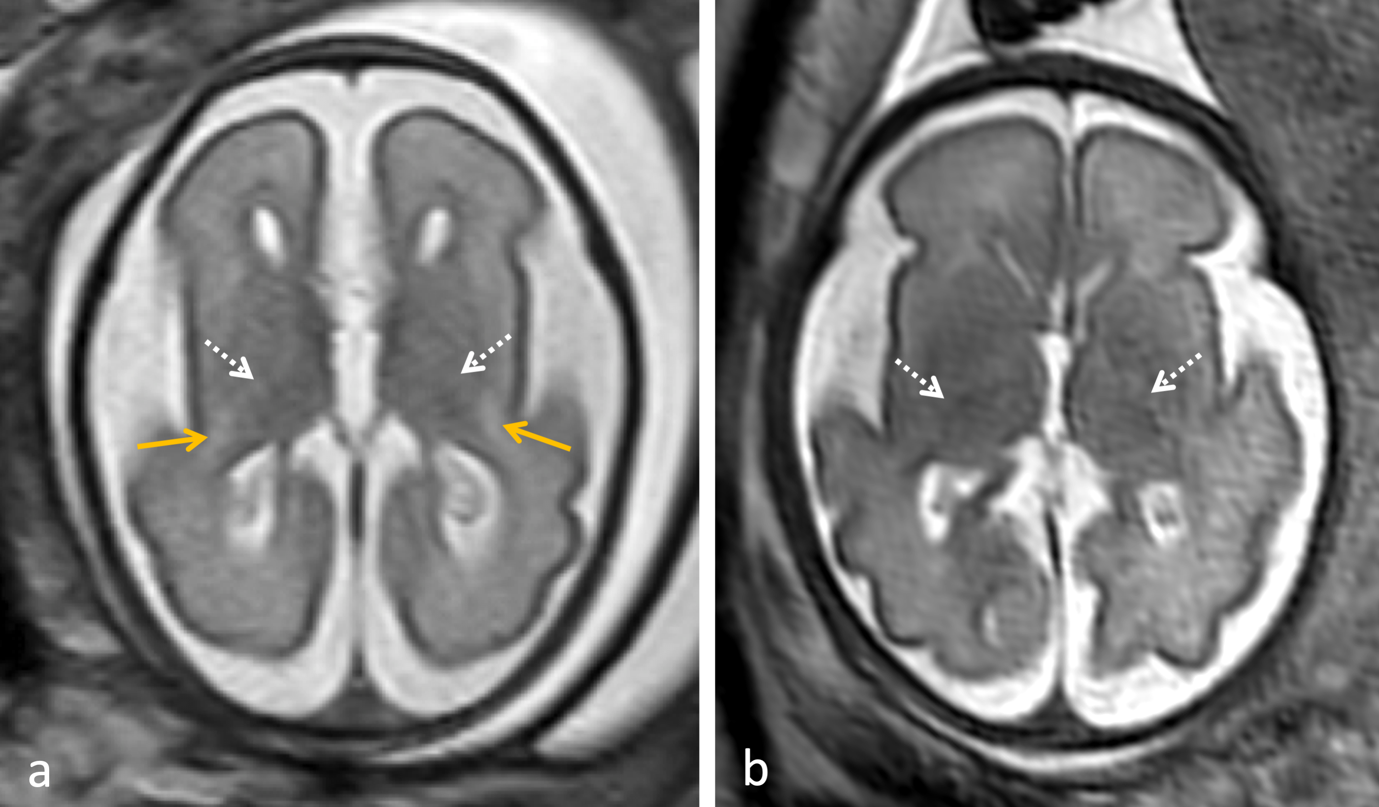
**

**Figure S8**


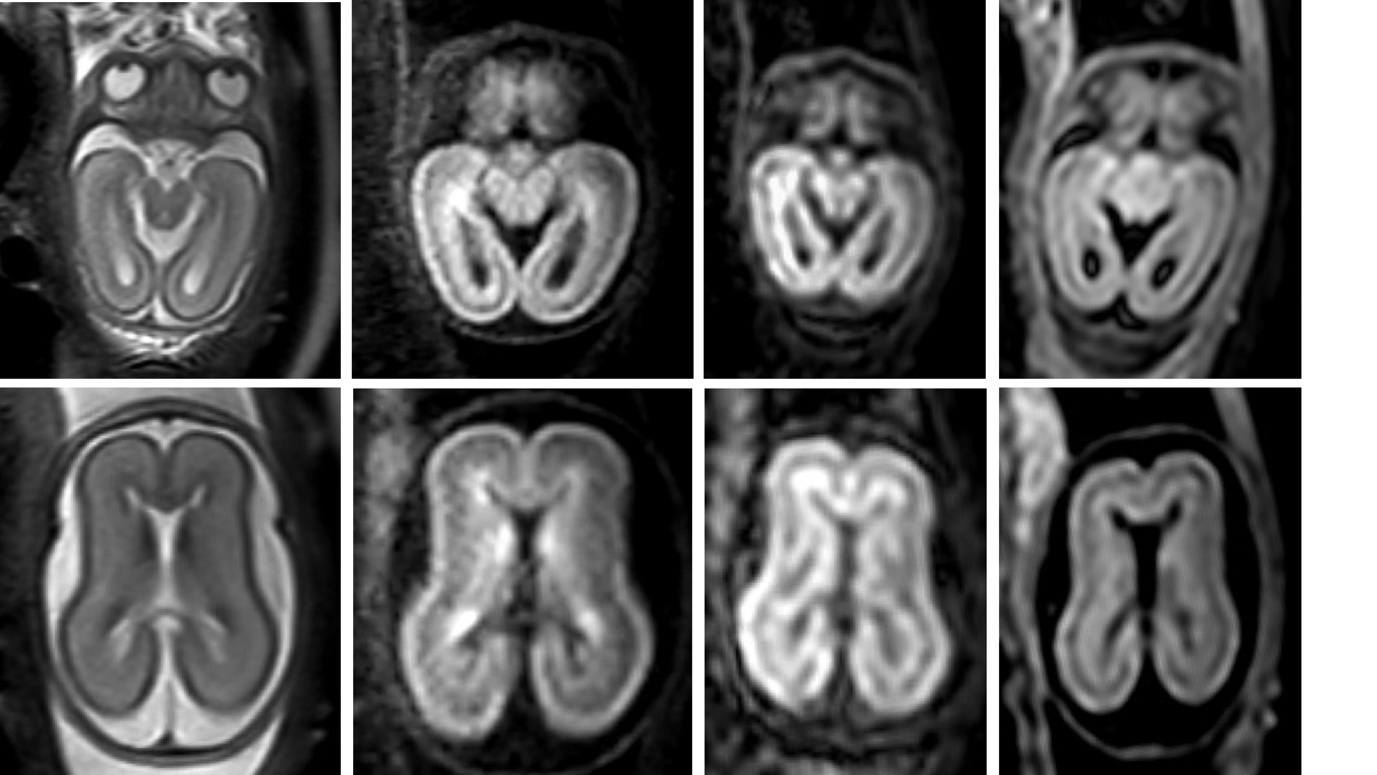


**Figure S9**
